# Brillouin Optical Correlation Domain Analysis in Composite Material Beams

**DOI:** 10.3390/s17102266

**Published:** 2017-10-02

**Authors:** Yonatan Stern, Yosef London, Eyal Preter, Yair Antman, Hilel Hagai Diamandi, Maayan Silbiger, Gadi Adler, Eyal Levenberg, Doron Shalev, Avi Zadok

**Affiliations:** 1 Faculty of Engineering and Institute for Nano-Technology and Advanced Materials (BINA), Bar-Ilan University, Ramat-Gan 5290002, Israel; sternyyy@gmail.com (Y.S.); yoseflondon@gmail.com (Y.L.); pre_eyal@yahoo.com (E.P.); antyai@gmail.com (Y.A.); hagaid@gmail.com (H.H.D.); 2 Xenom Ltd., 4 Gluska st., Rishon LeTzion 7565004, Israel; maayan.silbiger@gmail.com (M.S.); Gadi@xenom.com (G.A.); 3 Department of Civil Engineering, Technical University of Denmark, Kongens Lyngby 2800, Denmark; eylev@byg.dtu.dk; 4 Doron Shalev Engineering Ltd., 23 Bar-Kochba st., Bnei-Brak 5126002, Israel; doron@doron-eng.co.il

**Keywords:** structural health monitoring, optical fiber sensors, Brillouin sensors, composite materials, composite beams, strain measurements, Brillouin optical correlation domain analysis

## Abstract

Structural health monitoring is a critical requirement in many composites. Numerous monitoring strategies rely on measurements of temperature or strain (or both), however these are often restricted to point-sensing or to the coverage of small areas. Spatially-continuous data can be obtained with optical fiber sensors. In this work, we report high-resolution distributed Brillouin sensing over standard fibers that are embedded in composite structures. A phase-coded, Brillouin optical correlation domain analysis (B-OCDA) protocol was employed, with spatial resolution of 2 cm and sensitivity of 1 °K or 20 micro-strain. A portable measurement setup was designed and assembled on the premises of a composite structures manufacturer. The setup was successfully utilized in several structural health monitoring scenarios: (a) monitoring the production and curing of a composite beam over 60 h; (b) estimating the stiffness and Young’s modulus of a composite beam; and (c) distributed strain measurements across the surfaces of a model wing of an unmanned aerial vehicle. The measurements are supported by the predictions of structural analysis calculations. The results illustrate the potential added values of high-resolution, distributed Brillouin sensing in the structural health monitoring of composites.

## 1. Introduction

Composite materials are widely employed within critical structures in the aerospace, construction, automotive and healthcare sectors, due to their favorable ratio of strength to weight and the large freedom in the design of their mechanical properties and geometry. The potential consequences of failure in many composite material structures are catastrophic. Hence the structural health monitoring (SHM) of composites is often necessary at the design phase, during production, as part of in-service preventive maintenance procedures and in damage assessment [1,2]. Methods for SHM in composites include ultrasonic testing [1,2,3,4,5,6], thermography [7,8,9], and measurements of static deformation and dynamic vibrations [1,10,11,12,13,14]. Static strain and dynamic vibration analyses are often restricted to the reading of point-sensors, such as strain gauges, accelerometers [12] or non-contact laser Doppler vibrometers [1,14]. The scaling of these sensors to the continuous coverage of large areas is challenging or altogether impractical.

Optical fibers constitute an exceptional sensing platform [15,16]. They may be readily embedded within most structures with little effect on functionality, are immune to electro-magnetic interference, can be addressed from a long stand-off distance, and may be used in hazardous environments where electricity is forbidden. The most widely utilized fiber-based sensors in SHM are fiber Bragg gratings, which provide point-measurement of temperature, strain and vibrations [13,17,18]. As many as several hundreds of fiber Bragg gratings may be installed in large structures. However they cannot provide spatially-continuous readings.

Optical fibers also support distributed analysis protocols of both temperature and strain, based on the principles of Raman [19], Rayleigh [20] or Brillouin scattering [21,22], in which every section of the fiber itself serves as an independent sensing node. Coherent analysis of Rayleigh back-scatter was used in static strain measurements within the wing of an unmanned aerial vehicle (UAV) [23]. Distributed Brillouin sensors were introduced to SHM in aircrafts in 2001 [24], and to the analysis of large-scale composite structures in 2003 [25]. Brillouin sensing technology progressed significantly since these initial demonstrations, reaching a measurement range of 325 km [26], the addressing of over a million resolution points [27,28,29], acquisition rates of several kHz [30,31,32], and centimeter and even millimeter-scale spatial resolution [33,34,35]. These advances are being adapted in SHM applications in recent years, including in composites. Examples include modal vibrations analysis of panels with 20 cm resolution [36], Brillouin optical time-domain reflectometry with 10 cm resolution [37], and dynamic monitoring of steel beams [38] and composite strips [39]. However, in our opinion, the potential benefits offered by high-end distributed fiber sensing are not yet fully exploited in composites (and in SHM in general). 

In 2012, our group and coworkers proposed and demonstrated a Brillouin analysis protocol which allows for random-access monitoring of cm-scale segments within arbitrarily long fibers under test (FUTs) [40]. The protocol, known as phase-coded Brillouin optical correlation-domain analysis (or phase-coded B-OCDA), has since been extended to address over 400,000 resolution points [27,28]. Between 2014 and 2016, we had been working to implement phase-coded B-OCDA, with spatial resolution of 2 cm, over fibers embedded in composites. This paper summarizes the results of these efforts. A portable B-OCDA setup was designed and assembled on the production floor of a manufacturer of composite materials structures. Three different experiments are reported: the monitoring of epoxy resin curing, during and following the production of a composite beam; the estimate of bending stiffness and Young’s modulus of a composite beam based on distributed strain measurements in three-point bending tests; and the mapping of static strain in a loaded model of a composite UAV wing. Preliminary, partial results of our work were previously presented in conferences [41,42,43]. This paper brings together the outcome of the entire study, including previously unpublished results. The experiments illustrate the potential added values of high-resolution Brillouin analysis in the design, production and life-cycle monitoring of composites. 

## 2. Phase-Coded Brillouin Optical Correlation Domain Analysis 

Distributed Brillouin fiber sensors in general [21,22,44], and phase-coded B-OCDA in particular [27,28,40,45], are discussed in detail in many previous works. The measurement principles are presented here only briefly, for completeness. A comprehensive, recent review may be found in [46]. Stimulated Brillouin scattering (SBS) is a nonlinear optical interaction between pump and probe waves that counter-propagate along an optical fiber. The optical frequency of the pump wave is higher than that of the probe by a detuning ν, which is on the order of 10–11 GHz. The beating between the two optical waves stimulates a hyper-sonic acoustic wave of frequency ν, which co-propagates with the pump, through the mechanism of electrostriction [47]. The acoustic wave couples between the two optical fields due to the photo-elastic effect [47]. Coupling may lead to the amplification of the probe wave power, and to the attenuation of the pump. Effective stimulation of the acoustic wave requires, however, that the frequency detuning ν must agree with the Brillouin frequency shift (BFS) of the fiber $\nu_{B}$. The linewidth $\Delta \nu_{B}$ of SBS amplification is very narrow: only 30 MHz for continuous pump and probe waves. The exact value of the BFS varies with both temperature and strain. Therefore the mapping of the BFS is being used in the distributed analysis of both quantities.

Spatially-resolved Brillouin analysis mandates proper control over the location and timing of the acoustic wave stimulation. The protocol used in this work achieves such control through the joint phase-modulation of both pump and probe waves by a common, high-rate binary sequence. Consider a pump wave of fixed magnitude $A_{p0}$ that is launched into the fiber from z=0 and propagates in the positive z direction, and a counter-propagating probe wave of fixed magnitude $A_{s0}$ that enters the fiber at the opposite end z=L. Both waves are phase-modulated by the same sequence, so that the complex envelopes of the pump and probe at their respective points of entry may be expressed as [40,45]:

(1) $$A_{p}\left(t\geq 0\right)=A_{p0}u\left(t\right)=A_{p0}\underset{n}{\sum }c_{n}\mathrm{rect}\left(\frac{t-nT}{T}\right),$$

(2) $$A_{s}\left(t\geq 0\right)=A_{s0}u\left(t\right)=A_{s0}\underset{n}{\sum }c_{n}\mathrm{rect}\left(\frac{t-nT}{T}\right).$$

Here $\left\{c_{n}\right\}$ are the elements of a pseudo-random bit sequence (PRBS). Each element assumes a value of either 1 or −1 with equal probabilities. Also in Equations (1) and (2), T is the duration of each bit in the modulating sequence, rect(ξ)=1 for |ξ|≤0.5 and equals zero elsewhere, and t stands for time. Both $A_{p}\left(t\right)$ and $A_{s}\left(t\right)$ equal zero for t<0. The bit duration T is chosen to be much shorter than the acoustic lifetime $\tau =1/\left(2\pi \cdot \Delta \nu_{B}\right)$, which is on the order of 5–10 ns. Subject to the above modulation, one may show that the instantaneous complex magnitude of the stimulated acoustic wave at any point 0≤z≤L: Q(z,t), is given by [40,45]:

(3) $$Q\left(z,t\right)=jg_{1}A_{p0}A_{s0}^{*}\overset{t}{\underset{0}{\int }}exp\left[-\Gamma_{A}\cdot \left(t-t\prime \right)\right]\left\{u\left(t\prime -\frac{z}{v_{g}}\right)u^{*}\left[t\prime -\frac{z}{v_{g}}-\Theta \left(z\right)\right]\right\}dt\prime .$$

In Equation (3) $g_{1}$ is an electrostrictive parameter of the fiber, $v_{g}$ is the group velocity of light in the fiber, $\Gamma_{A}\left(z\right)\equiv 2\pi j\left(\nu_{B}^{2}-\nu^{2}-j\nu \cdot \Delta \nu_{B}\right)/\left(2\nu \right)$ is a complex linewidth, and $\Theta \left(z\right)\equiv \left(2z-L\right)/v_{g}$ denotes a position-dependent time lag. The expression suggests that the magnitude of the stimulated acoustic wave at a given z is closely associated with the auto-correlation function of the modulating waveform u(t), evaluated at a delay of Θ(z). For the phase modulation protocol of Equations (1) and (2), the correlation time of u(t) is approximately half the bit duration T. Hence the stimulation of the acoustic wave is effectively restricted to a narrow region of width $\Delta z=\left(v_{g}T\right)/2$, located at $z_{0}=L/2$ where $\Theta \left(z_{0}\right)=0$. At the correlation peak, $Q\left(z_{0},t\right)$ may build up to its steady-state value: $Q_{SS}\left(z_{0}\right)=\left(jg_{1}A_{p0}A_{s0}^{*}\right)/\Gamma_{A}\left(z_{0}\right)$. $Q_{SS}\left(z_{0}\right)$ reaches a maximum magnitude of $2jg_{1}A_{p0}A_{s0}^{*}\tau$ when $\nu =\nu_{B}\left(z_{0}\right)$ [45]. In all other locations z outside $z_{0}\pm \Delta z$, Q(z,t) rapidly oscillates about an expectation value of zero [45]. 

If the period of the sequence $\left\{c_{n}\right\}$ is sufficiently long, a single correlation peak is formed at $z_{0}$. In this case, measurements of the output probe power can be unambiguously related to the Brillouin amplification taking place at $z_{0}$. The measurement point may be scanned through the introduction of proper variable delays in the paths that lead the pump and probe waves into the FUT [34,35,40,44,45]. The Brillouin gain spectrum in each location is reconstructed through repeating acquisitions with different values of frequency detuning ν. More advanced variants of the phase-coded B-OCDA protocol support the simultaneous, unambiguous analysis of as many as 2000 measurement points in each trace [27,28,48,49]. However, these methods were not required in the analysis of the comparatively short fibers used in this work.

Since the local BFS changes with both temperature and strain, the analysis of the two effects using any Brillouin sensing protocol is inherently ambiguous. Several strategies were proposed for the separation of the two parameters: (a) installation of specialty fiber cables that provide thermal or mechanical isolation from the structure under test; (b) use of two dissimilar fibers in parallel; (c) measurement of additional optical properties, such as birefringence; or (d) prior knowledge of specific processes or conditions.

## 3. Measurement Setup

**Figure 1:**
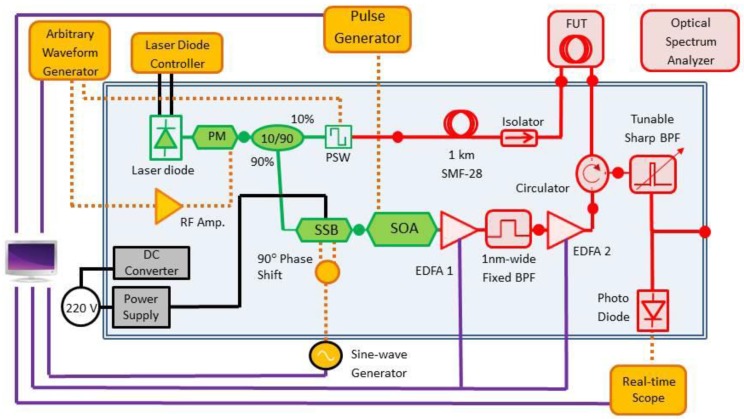
Schematic illustration of the measurement setup used in phase-coded Brillouin optical correlation domain analysis along fibers under test that were embedded in composite structures. Red, solid lines: standard single-mode fiber paths. Green, solid lines: polarization-maintaining fiber paths. Magenta, solid lines: instrumentation control lines. Orange, dashed lines: electrical radio-frequency signals paths. Black, solid lines: DC power supply lines. (Several additional power supply connections are not shown, for better clarity.) FUT: fiber under test. SOA: semiconductor optical amplifier. SSB: single-sideband electro-optic modulator. RF Amp.: radio-frequency amplifier. BPF: optical bandpass filter. EDFA: erbium-doped fiber amplifier. PSW: polarization switch. PM: electro-optic phase modulator. Closed circles represent input/output fiber connections for test-points or equipment interface.

A portable phase-coded B-OCDA measurement setup was designed and assembled. A detailed layout of the setup is shown in Figure 1. The setup consisted of a main box that contained all fiber-optic components, and standard laboratory equipment. Light from a laser diode was used as a common source for the pump and probe waves. The laser diode output passed through an electro-optic phase-modulator, which was driven by the output voltage of an arbitrary waveform generator. The generator was programmed to apply PRBSs with periods of 63 to 256 bits, and bit durations between 200–400 ps. These parameters correspond to B-OCDA spatial resolution of 2–4 cm, and separation of 1.25 m to 10 m between adjacent correlation peaks. The PRBS period was sufficiently long to guarantee that only a single correlation peak could form along the FUT. 

The phase-modulated waveform was divided in two arms. 90% of the intensity was directed to the pump branch. The frequency of the pump wave was up-shifted by an offset $\nu \approx \nu_{B}$ using a single-sideband electro-optic modulator, driven by the output voltage of a sine-wave generator. The amplitude of the pump wave was modulated by repeating, low-duty cycle pulses of 25 ns duration using a semiconductor optical amplifier. Last, the pump wave was amplified by two stages of erbium-doped fiber amplifiers to an average power of 200 mW, and routed into one end of the FUT through a circulator. The peak power levels of the pump pulses were several W.

Light at the 10% output of the coupler was used as a probe wave. The polarization of the probe was alternated between two orthogonal states, using a polarization switch driven by a square wave, to mitigate polarization-induced fading of SBS gain [50,51,52]. Parts of the setup were constructed of polarization-maintaining fibers, to support proper polarization switching (Figure 1). A fixed delay of 1 km of fiber was used for scanning the position of the correlation peak along the FUT (see [53] for details). The probe light at the output end of the FUT was detected by a photo-receiver, and the detector voltage was sampled by a real-time digitizing oscilloscope for further off-line processing. A narrow optical bandpass filter was used to block residual leaking of light at the pump wave frequency from reaching the detector. Measurements were taken at each position z along the FUT for several tens of detuning values ν, in steps of 1–3 MHz. The BFS at each z was identified through fitting the local measured Brillouin gain spectrum to a Lorentzian line shape of width $\Delta \nu_{B}$ [54]. The typical experimental uncertainty in the estimates of the local BFS values was ±1 MHz. This uncertainty corresponds to a temperature error of ±1 °K, or static strain error of ±20 µε. Standard FUTs with 250 µm dual-layer acrylate coating were used in all experiments. This type of fiber is the most readily available, and the simplest to connect with the measurement setup.

## 4. Experimental Results

### 4.1. Monitoring of a Composite Beam During and Following Production

The manufacturing of composites is accompanied by temperature elevation and the formation of strain, which are difficult to map using conventional SHM methods. In a first set of measurements, we have used the high-resolution B-OCDA setup for that purpose. Sensing optical fibers were installed below the outer-most sheet of structural glass-fibers within a one meter-long composite beam, prior to the application of epoxy resin. The width and height of the beam cross-section were 10 cm and 3 mm, respectively. Measurements were taken in twenty minutes intervals for more than sixty hours, with a resolution of 4 cm [41,42].

**Figure 2:**
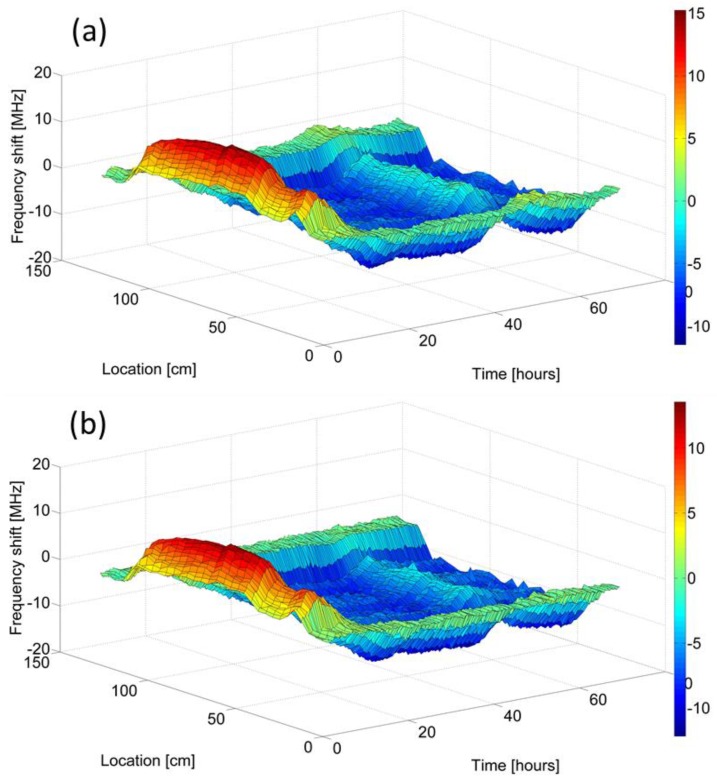
Measured Brillouin frequency shift as a function of position z along a fiber under test, and time elapsed since the installation of the fiber underneath the upper structural layer of a composite beam [41,42]. Phase-coded Brillouin optical correlation domain analysis was carried out at 20 min intervals for 60 h, during and following the production of the beam, with 4 cm resolution. (**a**): Offset of the Brillouin frequency shift from a nominal value of 10.85 GHz. (**b**): same as panel (a), following the subtraction of reference readings that were taken over the lead-in fibers outside the beam. The reference readings were updated once every 20 min. Variations in the laboratory temperature over the 60 h of measurements are therefore eliminated.

Figure 2a shows the measured BFS $\nu_{B}$, as a function of position and time elapsed since the fiber installation [41,42]. In Figure 2b, BFS values are presented as offsets from reference readings, which were taken over the lead-in fibers outside the beam once every twenty minutes. The lead-in fibers were strain-free, but their BFS drifted during the sixty hours of data acquisition due to changes in the laboratory temperature. These environmental drifts are subtracted out of the results of Figure 2b. 

The measurements show an initial increase of the BFS by approximately 15 MHz, which lasted for several hours. Since the FUT remains strain-free while the resin is still soft, the change in the BFS may be attributed to heating of the beam during the exothermic curing reaction. The measured change in the BFS corresponds to a temperature increase of about 13 °K [55]. The BFS values reached a steady state approximately twenty hours following the application of the resin. The steady-state BFS readings in fiber segments within the beam are lower than corresponding values over the lead-in fibers, by about 10 MHz. Since thermal equilibrium had been reached at the steady state, this offset indicates that a residual compressive strain of about 200 µε remains within the beam at the conclusion of the curing process [41,42]. The BFS of the fiber within the beam increased again, by approximately 5 MHz, about 50 h into the experiment. A smaller increase in BFS was observed along the lead-in fibers at the same time. The change was due to direct sunlight through the laboratory window in the afternoon hours of a particularly hot day. The resulting temperature increase within the composite beam was larger than that of the laboratory environment.

B-OCDA provided spatially and temporally continuous monitoring of the beam production process. The information obtained is difficult to achieve using standard SHM techniques, and may provide the manufacturer with effective quality control.

### 4.2. Measurement of Stiffness and Young’s Modulus in Three-Point Bending of a Composite Beam

**Figure 3:**
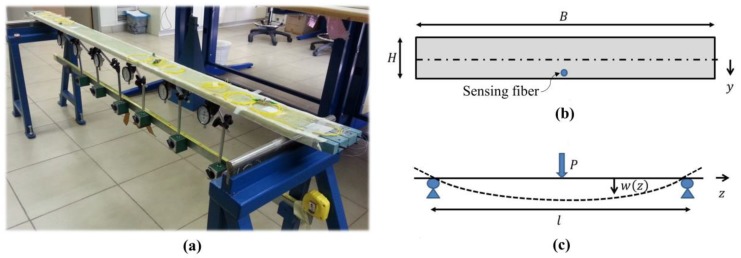
(**a**): A composite beam with embedded sensing fibers, placed in a three-point bending setup. The setup is equipped with six mechanical deflection indicators [42]. (**b**): Schematic illustration of the beam cross-section, showing the location of the sensing fiber. (**c**): An illustration of the bending configuration, with definition of the coordinate system used.

A two meter-long beam was produced for subsequent testing in a three-point bending setup [42]; it was comprised of structural layers of glass fibers and epoxy. The width and height of the beam cross-section were B= 12 cm and H= 2 cm, respectively. The sensing fiber was embedded underneath the outermost structural layer (see Figure 3b); the vertical offset distance of the sensing fiber from the centroid was y = 9.5 mm. A photograph of the test setup is presented in Figure 3a. In this picture the beam is seen to be simply supported at its edges by two cylindrical rollers, which were spaced apart by a length l= 1800 mm. Also shown in Figure 3c is a sketch of the setup, where w(z) denotes the deflection of the beam due to a force of magnitude P applied at the center of the span z=l/2. Moreover, six mechanical gauges were placed underneath the beam; these provided independent deflection measurements at discrete z positions.

The beam deflection at any point z is given by [56]:

(4) $$w\left(z\right)=\frac{P}{48EI}\left[3l^{2}z-4z^{3}+8{\left(z-\frac{l}{2}\right)}^{3}+8{\left|z-\frac{l}{2}\right|}^{3}\right],$$

where E is Young’s modulus and $I=\left(BH^{3}\right)/12=$ 8 × 10^−8^ m^4^ is the cross-sectional moment of inertia. The axial strain $\varepsilon_{z}\left(z\right)$ at an offset y is given by:

(5) $$\varepsilon_{z}\left(z\right)=\frac{y}{EI}\left[\frac{P}{2}z-\frac{P}{2}\left(z-\frac{l}{2}\right)-\frac{P}{2}\left|z-\frac{l}{2}\right|\right].$$

**Figure 4:**
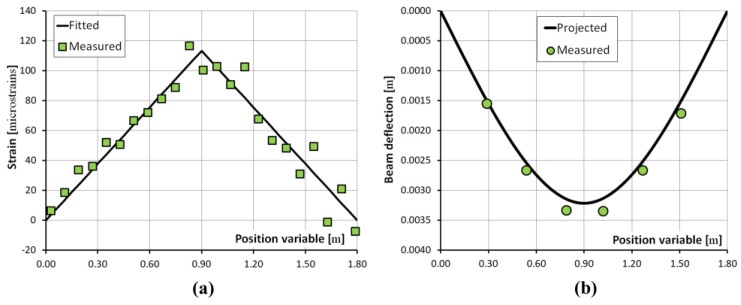
(**a**): B-OCDA strain measurements (squares), and computed strain after the calibration of bending stiffness (solid). (**b**): Independent point-measurements of beam deflection obtained with mechanical indicators (circles), and projected deflections based on the Brillouin analysis calibration of bending stiffness (solid) [42].

The strain in the bent beam along the embedded FUT was measured with the phase-coded B-OCDA setup [42]. Figure 4a shows the measured strain following the application of force P= 68.6 N. Data were used for fitting the bending stiffness of the beam EI= 2590 N·m^2^, using Equation (5) (see Figure 4a). The results suggest a value of E= 32.4 GPa for Young’s modulus of the glass-epoxy composite. Note that the scatter in measured strain is larger beyond the point of loading (z = 0.9 m). The calibrated modulus was used in forecasting the deflection profile of the beam, as in Equation (4). Figure 4b indicates excellent agreement between projected deflections and the readings of six independent mechanical gauges.

### 4.3. Strain Measurements in a Model Wing of an Unmanned Aerial Vehicle

**Figure 5:**
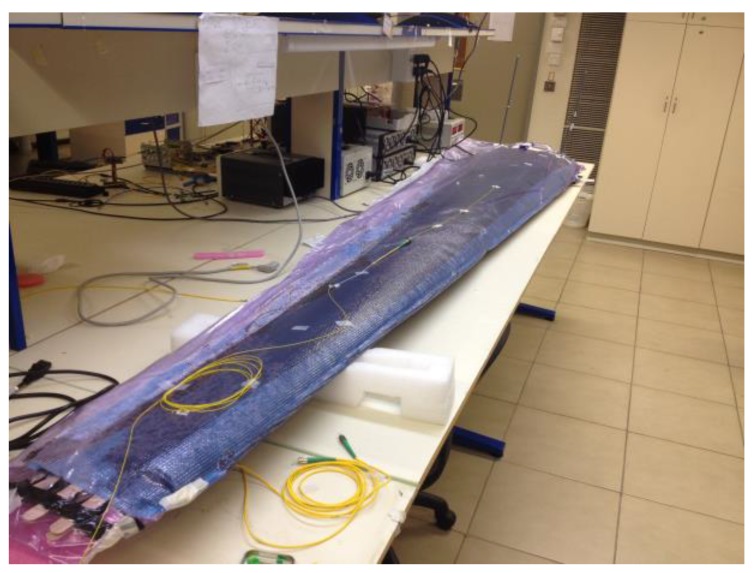
A model wing of an unmanned aerial vehicle, built with an outer skin of carbon-fiber and epoxy composite, with embedded sensing fibers.

In a final experiment, optical fibers were embedded within a model of a UAV wing [43]. The model was 2.5 m long. The loading of a solid wing would have required thousands of kg, therefore the wing model was built with a soft foam core covered by an outer composite skin. The fabrication of the model began with wrapping the compliant foam core with two sheets of carbon-fiber textile. Optical fibers were then laid along the upper and lower surfaces of the model, and held in place using contact adhesive. The ingress and egress points of the optical fibers were protected by hollow, 3 mm-diameter plastic tubes used in standard optical patch-chords, and fixed to wooden sticks. A third layer of carbon-fiber textile was wrapped on top of the sensing fibers, and the entire model was placed in a vacuum sack. Epoxy resin was pumped in to the sack and cured in vacuum [43]. Three optical fibers were embedded along the length of the lower surface of the model, and two more were installed in the upper surface. A third sensing fiber underneath the upper surface of the model broke during installation. No excess losses were observed in the remaining five FUTs. Figure 5 shows the wing model in the sensing laboratory.

The near edge of the model wing, (the one that connects with the body of the UAV), was rigidly clamped during testing. Different static loads were applied to the opposite, far edge. Reference values $\nu_{B}^{ref}\left(z\right)$ for the BFS along the sensing fibers were measured using phase-coded B-OCDA under self-loading of the beam, with no additional weight applied. The analysis was then repeated subject to static loads of 275 N (mass of 28 kg) and 550 N (56 kg). The additive strain due to loading was estimated based on the difference between $\nu_{B}\left(z\right)$ and $\nu_{B}^{ref}\left(z\right)$. The spatial resolution of the B-OCDA traces was 2 cm. The strain maps were averaged, however, by 10 cm-wide moving windows. 

**Figure 6:**
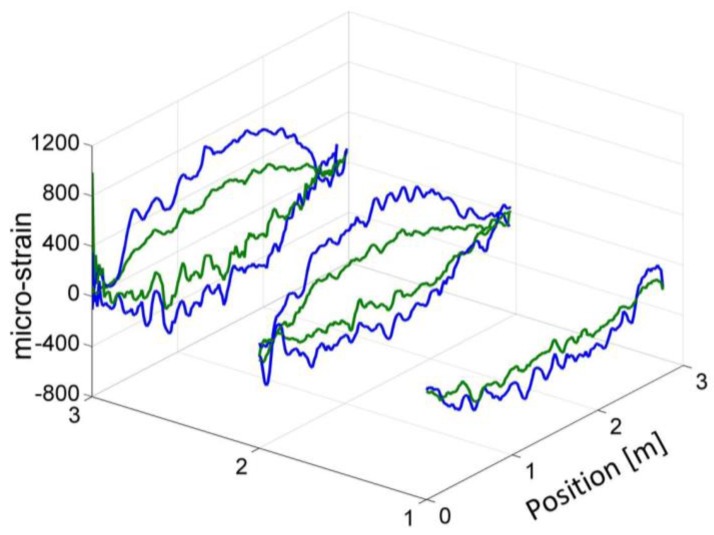
Strain variations due to the loading of a composite model wing of an unmanned aerial vehicle, measured using phase-coded B-OCDA. Five sensing fibers that were embedded in the outer skin of the model during production: two in the upper surface and three in the lower surface. Green and blue traces correspond to static loading of 275 N and 550 N, respectively.

Figure 6 shows the measured additive strain due to both loads, along the five embedded fiber segments. The added axial strain is seen to increase with load, as expected. The experimental uncertainty in the B-OCDA strain measurement, averaged over 10 cm, was only ±10 µε. We therefore suggest that the observed irregularities in the strain profiles represent ‘true’ variations, associated with the production and curing processes and with the manual installation of sensing fibers. 

**Figure 7:**
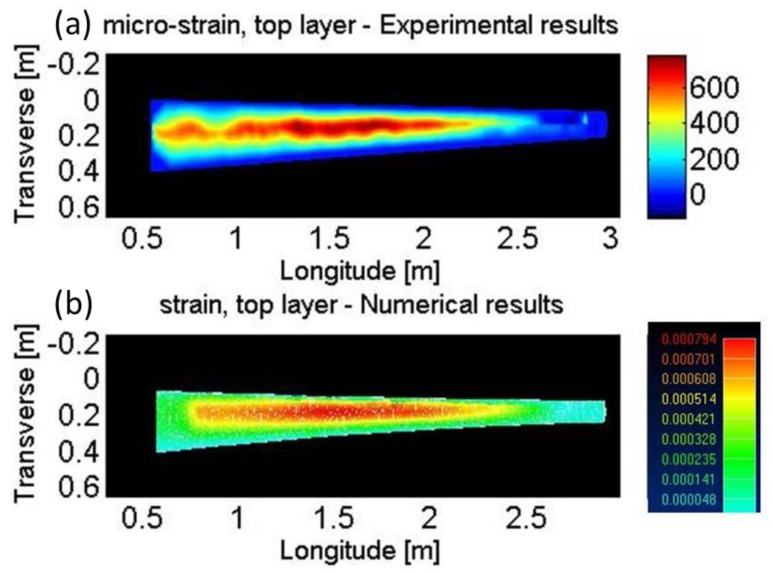
Strain profiles across the upper surface of a composite model wing of an unmanned aerial vehicle, subject to static loading of 550 N. (**a**): strain map extrapolated from high-resolution phase-coded B-OCDA traces. (**b**): predicted strain map obtained by numerical, structural analysis.

**Figure 8:**
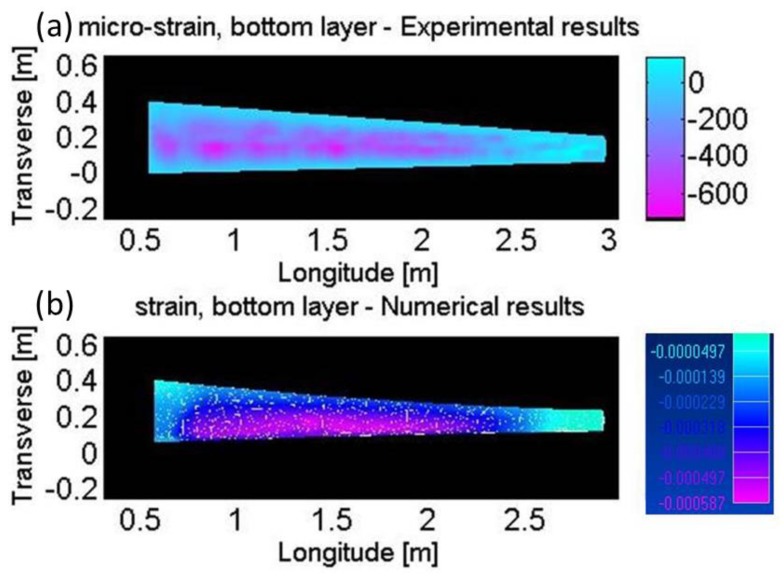
Strain profiles across the lower surface of a composite model wing of an unmanned aerial vehicle, subject to static loading of 550 N. (**a**): strain map extrapolated from high-resolution phase-coded B-OCDA traces. (**b**): predicted strain map obtained by numerical, structural analysis.

The strain measurements were compared against the predictions of numerical structural analysis, subject to 550 N load. Figure 7 and Figure 8 show the measured and calculated strain profiles across the upper and lower surfaces of the wing model, respectively. The two-dimensional, continuous experimental maps were obtained using cubic extrapolation between adjacent measurement points. Qualitative agreement is obtained between the predicted and measured strain maps. The order of magnitude of maximal strain, 800 µε, is in agreement as well. The detailed profiles in the two strain maps do not fully match, however. Potential causes for discrepancies include residual deviations between the manufactured model geometry and that of the design, imperfect clamping in the experimental test-bed, and residual strain induced during the fibers installation. Despite these limitations, the experiment provides a proof of concept for the application of high-resolution Brillouin analysis to a real-world composite model, outside the research laboratory. 

## 5. Conclusions

This work reported the application of high-resolution B-OCDA protocols to SHM of composites. The potential added values of the distributed analysis of temperature and/or static strain were demonstrated in three different experiments. First, the production and curing processes of a composite beam were continuously monitored over 60 h. The measurements revealed the heating profile that is associated with exothermic epoxy curing, and the residual compressive strain that remained in the beam at the conclusion of the process. Second, Young’s modulus of a composite beam was estimated based on high-resolution Brillouin analysis. The B-OCDA results were corroborated by independent measurements of beam deflection using six mechanical point-sensors. Last, strain profiles were mapped across the upper and lower surfaces of a model composite UAV wing, subject to static loading. Here too, the experimental strain maps were in general agreement with the predictions of structural analysis. The spatial resolution of the measurements reached 2 cm in several experiments. The experimental uncertainty in BFS measurements corresponds to errors on the order of ±1 °K or ±20 µε, or better. The analysis is scalable, in principle, to kilometers of fiber [27,28]. 

The measurements suffered from several drawbacks. The embedding of sensing fibers within the composite structures relied on manual installation, which is susceptible to local strain variations and is not repeatable. Installation was particularly challenging in the UAV wing model. Non-uniform, initial pre-strain in the sensing fibers introduces ambiguity in the interpretation of measured data. Better practices for the embedding of fibers would be required in future studies and applications. The Brillouin sensing configuration applied in this research is restricted to static analysis, due to the comparatively long acquisition durations of several minutes. This limitation, however, is not fundamental. As already noted in the introduction, measurement protocols for Brillouin sensing that are both of high resolution and dynamic have been proposed and demonstrated in other works [30,31,32,38,39]. Distributed fiber sensing is therefore applicable to vibration analysis of composites as well. Lastly, the size and weight of Brillouin sensing equipment currently prohibits the use of distributed analysis in many in-flight applications, particularly in UAVs. 

Despite the above restrictions, our results and those of other studies [23,24,25,36,37,38,39] successfully demonstrated that high-resolution Brillouin analysis of fibers embedded in composites may provide significant data that is difficult to obtain otherwise. Knowledge of spatially-continuous temperature and strain profiles can be highly instrumental in design validation and verification of scaled models; in quality control of production; in extraction of material parameters; in early detection of flaws during the life cycle of structures; and in failure analysis. The first three scenarios above were directly demonstrated in this work. This paper represents a significant step in the transition of high-end Brillouin sensing technology, from the fiber-optics laboratory towards potential field applications. Much work is still required towards this objective. However, the large potential benefits of distributed fiber sensing in SHM suggests that such efforts are likely to continue.
